# Respiratory Infections by HMPV and RSV Are Clinically Indistinguishable but Induce Different Host Response in Aged Individuals

**DOI:** 10.1371/journal.pone.0016314

**Published:** 2011-01-26

**Authors:** Vanessa Ditt, Jessica Lüsebrink, Ramona Liza Tillmann, Verena Schildgen, Oliver Schildgen

**Affiliations:** Institute for Virology, University of Bonn, Bonn, Germany; Institute of Infectious Disease and Molecular Medicine, South Africa

## Abstract

**Background:**

Human metapneumovirus and respiratory syncytial virus can cause severe respiratory diseases, especially in infants, young children, and the elderly. So far it remains unclear why infections in the elderly become life threatening despite the presence of neutralizing antibodies in the serum, and to which extent double infections worsen the clinical course.

**Methods:**

Young and aged BALB/c-mice were infected with RSV or/and HMPV. Appearance of the mice was observed during course of infection. On day 5 p.i. animals were dispatched by cervical dislocation and levels of TNF-α and NF-κB were determined.

**Results:**

The observation of activity, weight and appearance of the different mice showed no differences among the tested groups. Despite this, the immunologic response depends on the animals' age and the virus they were infected with. In young animals, NF-κB levels were elevated if infected with HMPV and HMPV/RSV but remained low in RSV infections, whereas in aged animals the opposite was observed: solely RSV-infected animals showed elevated levels of NF-κB. TNF-α was slightly elevated in HMPV-infected young and old animals, but only in young animals this elevation was significant.

**Conclusions:**

Contrary to other studies, no weight loss or change in activity despite productive lung infection with the different viruses were observed. This may be due to the weaker anaesthesia or the lesser volume of virus solution used, leading to less stress in the animals. The observed differences in TNF-α and NF-κB elevation lead to the assumption that young and old individuals have different mechanisms to react against the viruses.

## Introduction

In 2001 the human metapneumovirus (HMPV) was described as the third human-pathogenic member of the *Paramyxovirinae* besides respiratory syncytial virus (RSV) and parainfluenzaviruses (PIV) causing respiratory disease [1]. HMPV and RSV infections occur worldwide with a broad clinical spectrum from mild to severe and sometimes life threatening affection. Our group and others have shown that clinical severity of HMPV infections in hospitalized patients or in the elderly is indistinguishable from RSV infections on clinical grounds alone [2]–[8]. Nevertheless, despite those data, a still ongoing discussion that HMPV infections are generally milder survives obstinately. Surprisingly, thus far this latter question whether HMPV infections cause milder symptoms than RSV infections in otherwise healthy individuals has not yet been systematically addressed. Furthermore, HMPV and RSV are both frequently co-pathogens to each other and therefore may cross-react directly or indirectly during double infections [8], [9].

Genetically, HMPV is most closely related to the avian metapneumovirus (APV) [1] and is assumed to have a zoonotic origin as confirmed by the fact that it can be reversely transferred to poultry which is susceptible for APV. [10], [11], This zoonotic event must have occurred more than 50 years ago as shown by serological and bioinformatical analyses [1], [10].

In contrast to RSV, the HMPV genome lacks two genes coding for the non-structural proteins NS1 and NS2, both of which are assumed to interact with the host's immune response. For this reason RSV and HMPV may induce a different host immune response [12]–[18]. Comparative analysis of the cell-autonomous immunity against RSV and HMPV is hampered by a number of reasons, but recently we were able to show that the cell-autonomous innate immune response against both viruses is underlying different antidromic mechanisms [19].

Consequently, the first aim of our present study was to systematically investigate the natural course of RSV and HMPV mono- and co-infections in a murine model that emulates the infection in both risk groups, i.e. children and the elderly. Pilot studies in both age groups were performed previously by others but used comparatively high inoculation volumes of 50 to 150 µl [20]–[23]. Taking into account the total volume of a murine lung, which averages out at 500–700 µl with an average weight of approximately 500–700 mg in an adult mouse, these inoculation volumes appear to be too high to emulate the natural course of infection. Consequently we have reduced the inoculation volume to 25 µl per animal in order to simulate the conditions of natural host-to-host transmission. Surprisingly, despite of confirmed infection, all exposed animals displayed normal physiognomy and suffered only from mild disease, as described in clinical studies including otherwise healthy patients [2]–[8]. Furthermore, our investigation revealed that possibly a significant proportion of the so far published effects allocated to HMPV and/or RSV infection may have been induced by exposure to components related to the cell culture compounds of the inoculum. Finally, our data leads to the hypothesis that RSV and HMPV not only induce antidromic immune reactions in the individual age groups but that these effects invert in the aged animals, thus being double-antidromic with respect to age and virus specific immune response.

## Materials and Methods

### Ethics Statement

All animal experiments were performed according to national animal welfare regulations and with permission according to § 8 of the German Animal Welfare Act by the *Landesamt für Natur, Umwelt und Verbraucherschutz Nordrhein-Westfalen* (No. 9.93.2.10.35.07.143), i.e. the local and regional animal care commission.

### Cell Culturing

Human hepatoma HepG2 cells (ATCC HB-8065) were kindly provided by Dr. Ulrike Protzer (LMU/GSF Munich, Germany) and were maintained as previously described [24] in Dulbecco's modified Eagle's medium supplemented with 10% FCS, 2 mM L-glutamine, 50 U of penicillin/ml, 50 µg of streptomycin/ml, 1 mM sodium pyruvate, and nonessential amino acids (Invitrogen/Gibco, Karlsruhe, Germany).

### Virus strains and mock controls

The RSV isolate [25] and the HMPV strain HMPV-97/83 (kindly provided by Dr. Guy Boivin, Canada) were propagated as previously described on HepG2 cells [19]. Viruses were grown to high titers that were used for inoculation of mice. Inoculations were performed with 25 µl of virus suspensions with 2×10^7^ geq. The control medium was treated exactly as virus cell culture fluid, i.e. it was mounted for the same time on cells before being harvested and cleared by centrifugation. This mock control corresponds to the mock control proposed by Darniot and colleagues who named this mock control “virus free cell preparation” (6). PBS was used as additional control.

### Virus Stocks and Quantification

Light cycler real time RT-PCR was utilized to quantify the RNA content of HMPV and RSV-RNA stock solutions and virus preparations from infected HepG2 cells and supernatants and from infected lung tissues as previously described [26]. For quantification of RSV in analogy real time RT-PCR was performed using the primers RS-F1-LC 5′- AAC AGA TGT AAG CAG CTC CGT TAT C- 3′and RS-F2-LC5′- CGA TTT TTA TTG GAT GCT GTA CAT TT- 3′ at 50°C 20′; 95°C 15′; 45 cycles 95°C 20″, 58°C 60″, 72°C 30″; melting curve 95°C, 60°C (20°C transition rate, each), 90°C (0.05°C transition rate), using the SYBR Green One Step Real Time RT Kit from Qiagen (Hilden, Germany) according to the manufacturer's recommendations. Additionally, TCID_50_ for RSV and HMPV was determined respectively using plaque titration assays in 96 well format followed by crystal violet staining essentially as described and shown to be equivalent previously [27].

### Animals and in vivo infection

Four to six weeks old and 19 months old male BALB/c mice (inbred strain BALB/cJ_Rj) were purchased from Elevage-Janvier (Le Genest St. Isle, France). Mice were separated and kept in cages with activity wheels (Sandown Scientific, Hampton Middlesex, UK) voluntarily used by the animals. Animals were infected with 2×10^7^ genome equivalents (geq) HMPV, 2×10^7^ geq RSV, or double-infected with 1×10^7^ geq HMPV and RSV each, respectively, in a total volume of 25 µl cell culture supernatant clarified from cell debris by 10 min centrifugation at 2.000 g. Mock infections were performed with supernatant from mock infected cells harvested 5 days post mock infection and clarified from cell debris by 10 min centrifugation at 2.000× g. Further controls as “PBS mock” infection and “narcotized only” were included. Furthermore we infected two 4 weeks old male C57BL/6J_Rj and two 18 months old male C57BL/6J_Rj mice (Elevage-Janvier, Le Genest St. Isle, France) in order to test the susceptibility of this mouse strain for HMPV. All virus inoculations were performed intranasal under mild and flat inhalation anaesthesia with isofluorane. Physiognomy, body weight, food consumption, and activity were measured during a three day cage/activity wheel adaption phase, a five day pre-infection phase, and a five day post-inoculation phase. Thereby we made use of activity wheels that can be used *ad libitum* by the animals. Food and water were also fed *ad libitum* and the average food consumption per day was controlled by regularly weighing of the non-consumed food. Five days after inoculation all animals were dispatched by cervical dislocation and lungs were prepared. Lung tissue was weighted and homogenized in PBS in equal w/v ratios using a Qiagen TissueLyser (Hilden, Germany). Portions of the lung homogenates were used for determination of collagen, TNF-α, and for extraction of viral RNA. RNA extractions were again performed with the Qiagen RNeasy Mini Kit according to the manufacturer's protocol, as described above.

### TNF-α and NF-κB ELISA

ELISA assays for murine and human TNF-α and NF-κBp65 were purchased from Invitrogen (Karlsruhe, Germany). Murine lung tissue homogenates (see above) from the different infection groups (HMPV, RSV, HMPV/RSV) respectively, as well as from the control groups (only anesthetised, untreated animals) also including mock infection with exhausted cell culture supernatant or with PBS, respectively, were pooled, serially diluted, and analysed in triplicate against internal test controls. Initially, lung tissue was homogenized in PBS and lysed with lysis buffer (20 mM Tris pH 7,4, 140 mM NaCl, 10 mM NaF, 10 mM Natriumpyrophosphat x10·H_2_O, 0,1% Triton X-100, 1 mM EDTA, 1 mM EGTA, 1 mM Natriumorthovanadat, 20 mM β-Glycerophosphat). Protein concentration was estimated with the Quick Start™ Bradford Dye Reagent (Bio-Rad, München, Germany). Equal amounts of protein were pooled and analyzed in each assay. Experiments were performed at least in triplicate.

### Collagen assay

The amount of soluble collagens was estimated with the Sircol-Assay (Tebu-Bio, Offenbach, Germany) in line with the included manual.

## Results

The aim of the study was to systematically analyze and compare the outcome of RSV, HMPV, and RSV/HMPV co-infections in otherwise healthy young and elderly individuals in an animal model. Thereby a prerequisite of the study was to emulate the natural infection as realistic as possible. In contrast to other relevant studies, the inoculation volume was reduced to 25 µl containing 2×10^7^ geq in order to avoid large liquid volumes in the lung of the inoculated animals and to generate an infection starting in the upper airway before manifestation in the lower respiratory tract. For this reasons, 2–4 weeks and 19 months old BALB/c mice were infected with 2×10^7^ geq hRSV or hMPV, or co-infected with 1×10^7^ geq of each virus, respectively. Thereby it is important to keep in mind that it was shown previously for HMPV and RSV that the genome equivalents determined by real-time RT-PCR are equivalent to TCID_50_ using plaque titration assays in 96 well format followed by crystal violet staining essentially and real time cell electronic sensing as shown previously [27]. Untreated animals, animals anesthetized and treated with cell culture supernatant or PBS, or solely anesthetized animals served as controls. The additional controls served as verification, if observed differences between the different animal-groups were virus specific.

### Physiognomic Investigations of Infected Animals

**Figure 1:**
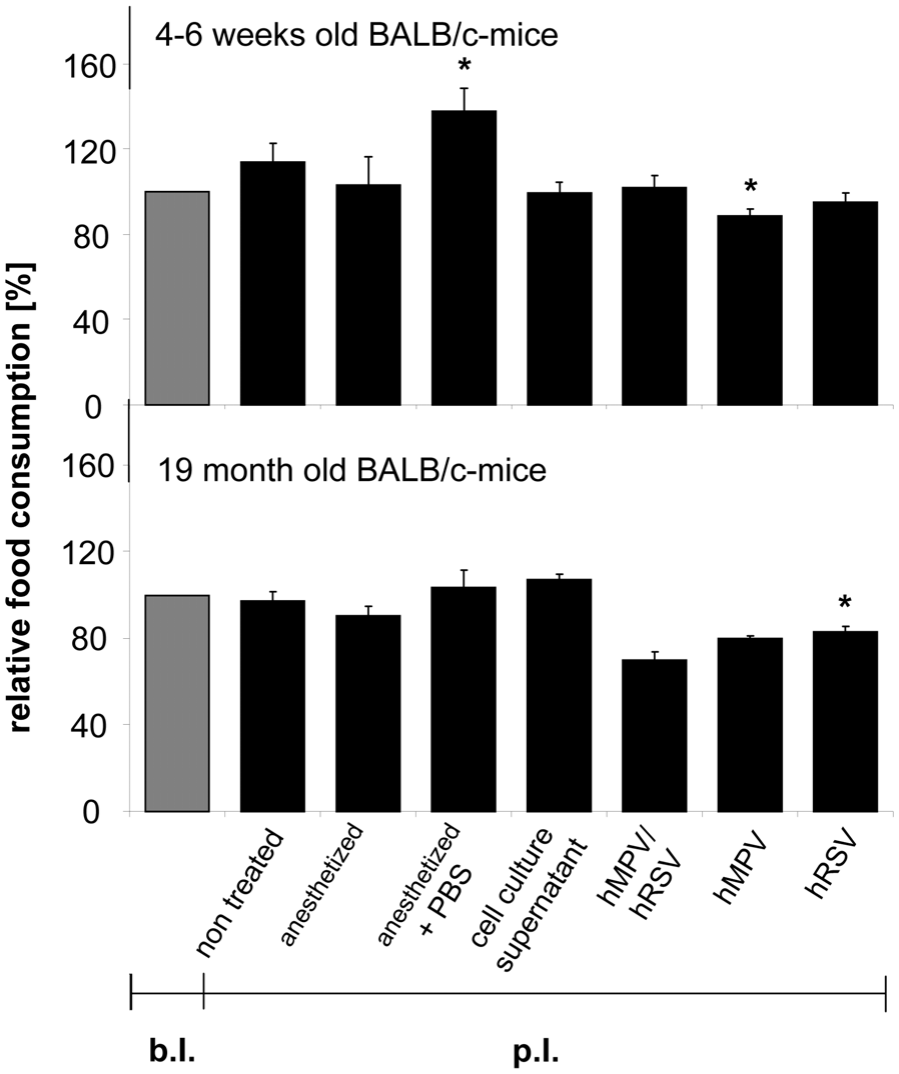
Daily food consumption of infected animals. Daily food-consumption of 4–6 weeks (A) and 19 month (B) old BALB/c mice before and after infection with RSV, HMPV or double infection: 4–6 weeks and 19 months old BALB/c mice were infected with 2×10^7^ geq RSV or HMPV, or co-infected with 1×10^7^ geq of each virus. Untreated animals, animals anesthetized and treated with cell culture supernatant or PBS or only anesthetized served as controls. The food consumption was recorded at the beginning of the experiment, on the day of inoculation and at the end of the experiment. Values were standardized referring to the values before infection (n = 3–5, values are shown as mean ± standard deviation). + significanty different to the corresponding value before infection (b.i.) (p<0.05).

**Figure 2:**
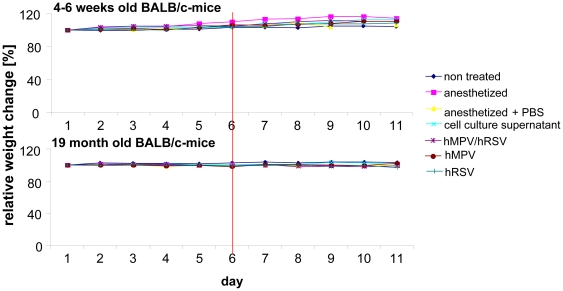
Weight change of infected animals. Weight change of 4–6 weeks and 19 month old BALB/c-mice before and after infection with RSV, HMPV or double infection: 4–6 weeks and 19 months old BALB/c mice were infected with 2×10^7^ geq RSV or HMPV, or co-infected with 1×10^7^ geq of each virus. Untreated animals, animals anesthetized and treated with cell culture supernatant or PBS or only anesthetized served as controls. Animals were weighed daily. All values were set in relation to the weight of the animals on day 1. The red line marks the day of infection (n = 5, values are shown as mean).

Animals were kept in separate cages with free access to an exercise wheel, which allowed measurement of the activity. Weight, physiognomy, food consumption, and activity were checked and documented daily. No differences in weight changes (figure 1), activity, or appearance of the animals' fur (data not shown) were observed, thus there were no physiognomic signs of the infections as described by others. Moreover, no weight loss was observed during the entire observation period neither in young nor in aged animals (figure 2).

### Viral genome replication in infected animals

**Figure 3:**
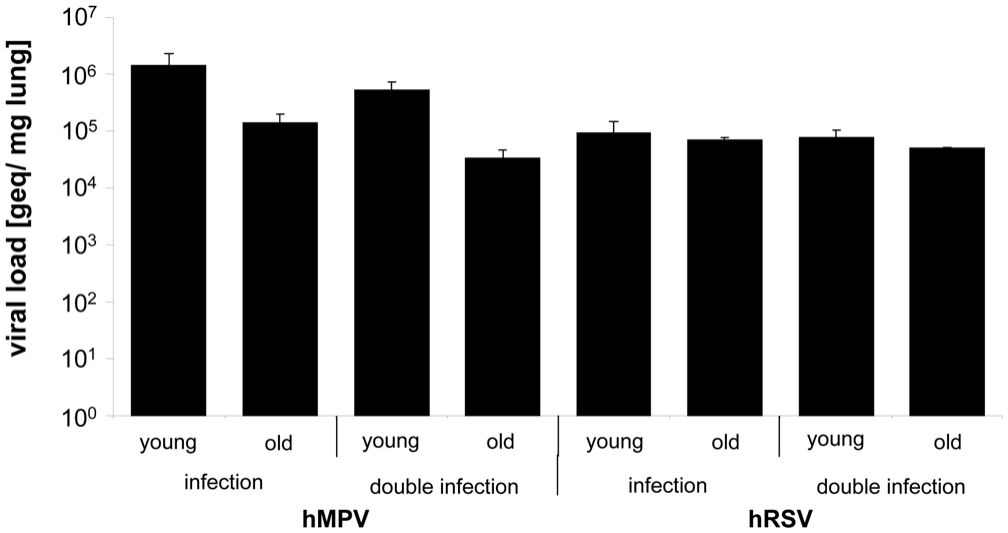
Viral load of lungs after infection. Viral load of lungs obtained from 4–6 weeks and 19 months old BALB/c mice after infection with RSV, HMPV or double infection: 4–6 weeks and 19 months old BALB/c mice were infected with 2×10^7^ geq RSV or HMPV, or co-infected with 1×10^7^ geq of each virus. Untreated animals, animals anesthetized and treated with cell culture supernatant or PBS or only anesthetized served as controls. Mice were killed by cervical dislocation and the lungs were removed, homogenised and viral RNA was extracted. The number of genome equivalents was estimated by qRT-PCR (n = 3–5, values are shown as mean ± standard deviation).

Although the lack of physiognomic changes of the animals could be interpreted as an absence of a productive infection in the first instance, the analyses of viral titres in lung tissues revealed that both viruses reached the lung tissue as in natural infections: Both viruses replicated to titres of up to >10^6^ geq/ml at day 5 post infection, whereas no virus was detected in the control animals (figure 3). Thereby the lung of a mouse has an average weight of ∼0.5 g, i.e. 500 mg. The inoculated amount of virus was 2×10^7^ geq which corresponds to approximately 4×10^4^ geq per mg lung. We detected ≥10^5^ geq at day five post infection, thus the inoculated amount was at least duplicated; consequently it has to be assumed that replication took place. Viral replication was also observed in C57BL/6 mice, of which 2 young and two adult mice were infected (data not shown).

### Measurement of expression of TNF-α and total NF-κB p65

**Figure 4:**
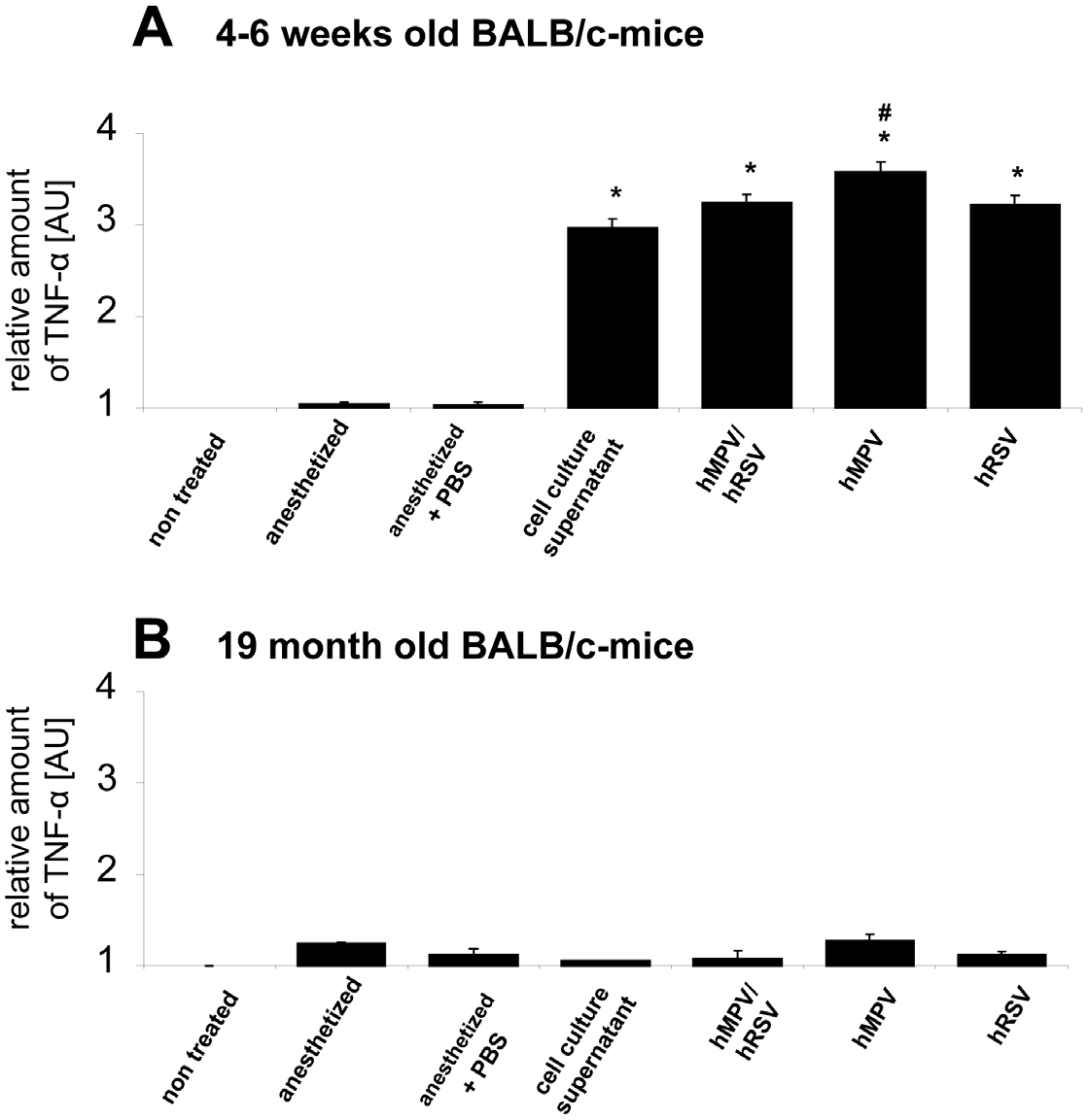
TNF-α expression in the lung after infection. Amount of TNF-α in the lungs of 4–6 weeks (A) und 19 months (B) old BALB/c mice after infection with RSV, HMPV or double infection: 4–6 weeks and 19 months old BALB/c mice were infected with 2×10^7^ geq RSV or HMPV, or co-infected with 1×10^7^ geq of each virus. Untreated animals, animals anesthetized and treated with cell culture supernatant or PBS or only anesthetized served as controls. Mice were killed by cervical dislocation and the lungs were removed and homogenised. The amount of TNF-α was determined by ELISA. Values were standardized referring to non treated controls (n = 3–5, values are shown as mean ± standard deviation). * significantly different to untreated animals (p<0.05). # signifcantly different to cell culture infected animals (p<0.05).

If compared to the mock controls the virus infections in turn led to significant increases of TNF-α in young animals independent of the virus the animals were infected with (figure 4A). Most surprisingly, a very similar increase in TNF-α expression was observed if animals were mock infected with depleted cell culture supernatant, indicating a virus independent mechanism of TNF-α activation induced by cellular metabolites that remained in solution whilst the cell supernatant was subject to centrifugation at 2000× g. Solely the HMPV infection led to a TNF-α increase which is statistically significant higher than the effect induced by the depleted cell culture medium. Additionally, only very moderate increases in the expression of TNF- α were observed in the aged mice (figure 4B), leading to the hypothesis that in those animals naïve for HMPV and RSV, the adaptive innate immune response was weak; as the animals were kept pathogen free before getting included in the experiments an infectious component of immunosenescence in the present study is rather unlikely and may be of importance as an additional factor in humans who acquired various pathogens during their lifespan.

**Figure 5:**
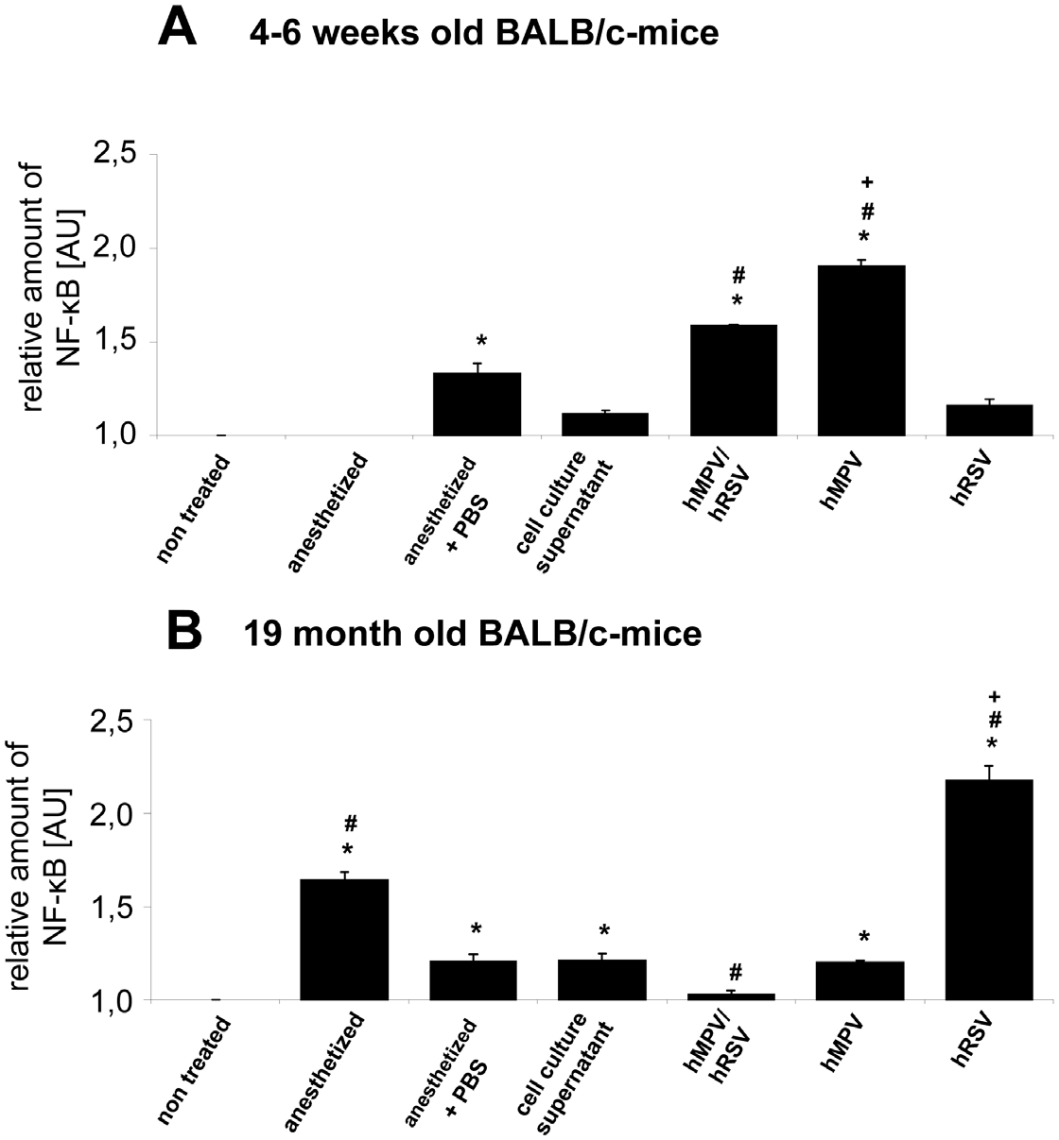
NF-κB expression in the lung after infection. Amount of NF-κB in the lungs of 4–6 weeks (A) und 19 months (B) old BALB/c mice after infection with RSV, HMPV or double infection: 4–6 weeks and 19 months old BALB/c-mice were infected with 2×10^7^ geq RSV, or HMPV, or co-infected with 1×10^7^ geq of each virus. Untreated animals, animals anesthetized and treated with cell culture supernatant or PBS or only anesthetized served as controls. Mice were killed by cervical dislocation and the lungs were removed, homogenised. The amount of NF-κB was determined by ELISA. Values were standardized referring to non treated controls (n = 3–5, values are shown as mean ± standard deviation). * significantly different to untreated animals (p<0.05). # signifcantly different to cell culture infected animals (p<0.05). + significantly different to hMPV and hMPV/RSV-infected animals (p<0.05).

The increased amounts of TNF-α induced the expression of activated NF-κB (figure 5). Thereby it appears that in young animals HMPV induces a high NF-κB level whereas RSV induces low levels; in the aged animals the opposite effect was observed, indicating that both viruses induce a virus-type specific effect that underlies additional, so far unknown, immune mechanisms which are age-dependent (figure 5A and B). In young animals the double infection induces lower NF-κB levels than the HMPV but higher levels than the RSV mono-infections, whereas in aged animals nearly no NF-κB was detected in this group. Surprisingly, as an incidental background finding, in aged animals the isofluorane anaesthesia led to a significant increase in the NF-κB levels which was not observed if any liquid was applied to the animals airways after anaesthesia. This may indicate that the pure inhalation of isofluorane may have a toxic component that needs to be taken into account in any future study. Most surprisingly, no similar effect was observed in the young mice and no literature data are available on this effect so far. It was technically impossible due to the overall setting of the present study to analyse NF-κB translocation to the nucleus, but the fact that NF-κB expression itself is misregulated as a consequence of increased TNF-α expression adds value to the present study. Future research has to focus on these aspects, and investigate how far the increase in NF-κB expression is followed by increased translocation of NF-κB subdomains to the nucleus.

### Measurement of Collagen Expression

**Figure 6:**
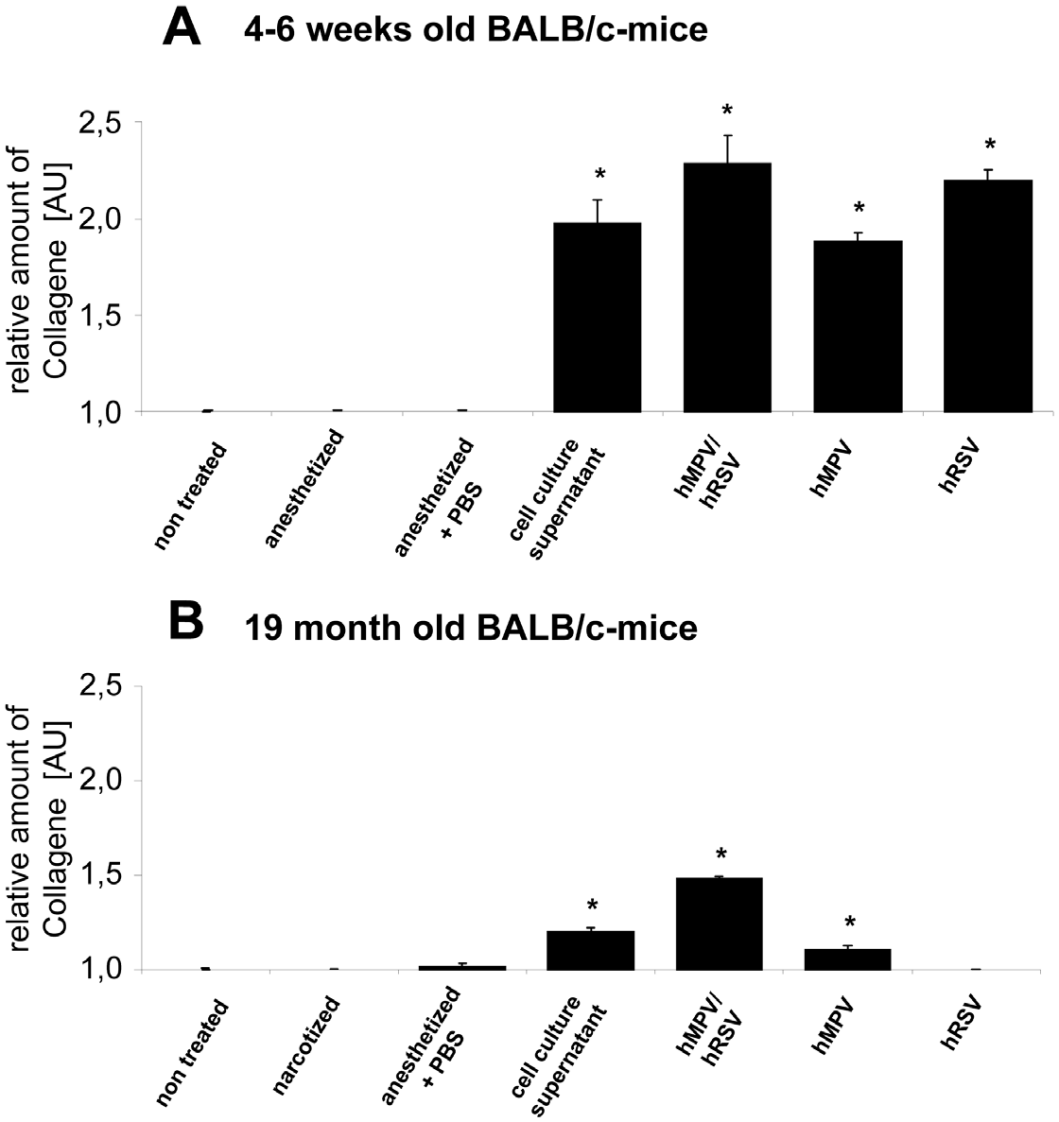
Soluble Collagen Expression in the infected lung. Amount of soluble collagens in the lungs of 4–6 weeks (A) und 19 months (B) old BALB/c mice after infection with RSV, HMPV or double infection: 4–6 weeks and 19 months old BALB/c mice were infected with 2×10^7^ geq RSV, or HMPV, or co-infected with 1×10^7^ geq of each virus. Untreated animals, animals anesthetized and treated with cell culture supernatant or PBS or only anesthetized served as controls. Mice were killed by cervical dislocation and the lungs were removed and homogenised. The amount of soluble collagens was determined by a collagen assay. Values were standardized referring to non treated controls (n = 3–5, values are shown as mean ± standard deviation). * significantly different to untreated animals (p<0.05).

Finally we addressed the question to which extent expression of proteins of the extracellular matrix is induced by the infections with either of both pathogens. This question is important as an increase of those proteins on the one hand may contribute to the pathogenesis of subsequent asthma, a condition known to be induced by respiratory viruses in general and HMPV and RSV in particular. On the other hand, expression of extracellular matrix proteins is a marker of inflammation, too, as immune cells need to attach to matrix proteins whilst moving themselves to the site of infection in the lung. In fact, despite a general cell culture induced effect, the double infection with HMPV and RSV tends to induce a higher expression of collagens in the lung of both young and old animals (figure 6). Although the data are not statistically significant they are direction-giving as the used assay detects the soluble collagens (i.e. collagens I-V) and cannot differentiate between the different collagen subforms; however, previous cell culture data [19] confirm the observation that collagen plays a putative role in HMPV and RSV infections.

### Results summary

In otherwise healthy individuals the primary infection with RSV, HMPV or both viruses simultaneously leads to modest TNF-α response, modest NF-κB response and virtually no collagen response, the latter in turn indicating that airway remodelling and subsequent asthma is dependent on additional factors. Surprisingly, HMPV and RSV induce an age dependent cross-over effect that is characterized by a virus specific component but is independent of infectious immunosenescence.

## Discussion

Respiratory infections remain a major global health burden that especially affect high risk groups like immunosuppressed, young children and the elderly; in the latter cohort of patient they are the fourth most common cause of death. HMPV and RSV are members of the Paramyxovirus family and have been associated with mild to severe respiratory infections. Life threatening infections were observed for both viruses but are mainly associated with diverse underlying diseases that contributed to the severe clinical course. However, it remained under discussion that RSV is a more severe pathogen than HMPV, but systematic studies on this assumption were missing. Furthermore it becomes more and more clear that especially viral respiratory infections directly or indirectly contribute to fatal infections in the elderly and are the 4^th^ most frequent cause of death in the this age group.

Genetically, both viruses share large similarities in their genomes, but RSV has two additional genes, namely NS1 and NS2 that were shown to act as immunomodulators which interact and interfere with multiple parts of the interferon and STAT2 pathways [13], [28]–[30].

Most interestingly, Bitko and coworkers [31] found that the RSV NS proteins suppress apoptosis by a NF-κB dependent mechanism; this finding is surprising as NF-κB is induced solely in the aged RSV infected mice but not in the young RSV infected mice, whereas the effect with HMPV is exactly the opposite. The induction of NF-κB in case of RSV infections is moreover independent of TNF-α. In the present study, however the expression of total NF-κB is likely to be induced by the high TNF-alpha levels in the young HMPV infected mice. Both results, the NF-κB levels and the TNF-α levels in young mice confirm previous results by Huck and colleagues who observed that RSV infection induces an innate immune response that differs from HMPV infection. The data also partially confirm the recent French study by Darniot and coworkers who investigated the immunological response in aged mice against HMPV [32]. In both previous studies and other studies animals were inoculated with volumes of 50–150 µl of virus suspension which in turn is up to one third of the animals lung volume.

We reduced the volume to 25 µl in order to avoid swallowing of the inoculum and to preserve the animal's lung from being overwhelmed with liquid. In turn we did not observe physiognomic changes as the other studies do, most likely as the deeper anaesthesia and the higher volume lead to malaise and fatigue in the animals anesthetised with Ketamin and inoculated with larger volumes. Taking into account the successful infection of the lung, the presented changes in the technical procedures therefore are an improvement to emulate a more natural infection, as the estimated course of infection takes place by low inoculation volumes of smear and droplets. Furthermore, with respect to age-related aspects during the respiratory infection with HMPV and RSV the C57BL/6 mice could be a useful model that is widely commercially accessible and required less institutional infrastructure for in-house animal-housing during the animals' lifespan.

In addition, another observation deserves foremost attention. The negative control used in this study was depleted cell culture fluid mounted to HepG2 cell for the same time as the virus was grown in those cells; the supernatant was cleared by centrifugation in order to get rid of cellular debris – the same was done with the virus stocks – but still may contain soluble proteins that may have an immunomodulatory effect. This mock control is exactly the kind of control that was also used by Darniot and coworkers (6) who contrary to our group made use of larger inoculation volumes (150 µl). Such an appropriate control was missing in most RSV and HMPV studies published so far who made use of PBS, fresh cell culture media or relinquished on mock infection in total whilst solely using non-infected animals, thus it remains unclear which effects are true virus induced effects and which not. Nevertheless, the remaining effects observed in the present study are congruent with the observations by Huck, Alvarez, and Darniot but also go beyond these previous studies [32]–[35]. This study shows for the first time that both infections are indeed mild in otherwise healthy individuals. Furthermore it demonstrates that the double infection with RSV and HMPV is a true intermediate between both single infections. As the effect observed in the present study is less pronounced than in the study by Darniot *et al.* (6), it may be worth to setup a study that focuses on the effect of the inoculation volume on the physiognomic and immunological outcome of the infection. Furthermore it would be worth to add gamma irradiation inactivated virus as an additional control which was impossible in the present study due to infrastructural reasons.

Moreover it is worth to mention that we observed an increase in NF-κB expressionin all mice with anaesthesia but not in mice with anaesthesia plus PBS. This result underlines the effects liquids exert on the animals' airways. It can be easily imagined that the inhalation of isofluorane leads to airway irritation that in turn is compensated by the rinsing with PBS. Consequently we conclude that this effect has to be taken into account in any future study.

As the most important observation of the present study it became clear that both viruses induce different innate immune reactions that have an additional age related component. Most interestingly, both responses appear to be the opposite of each other and change cross-over by age. Therefore further studies are required to elucidate the role of the individual pathogens but also copathogens in any age group as they cannot be easily compared. One of the co-pathogens that may additionally influence the outcome of the immune reaction is CMV, a herpes virus that persists lifelong and that is acquired by virtually all humans during their lifespan [36], [37]. However, CMV was not present in our model and thus may be rather an additional complication in the complex process of immune-aging that per se may influence the host response to a given pathogen.

Taking into account the fact that the major genetic difference between RSV and HMPV is the lack of NS proteins in case of HMPV, it is worth to generate an HMPV recombinant carrying these genes and to investigate their role in the double infection. Those studies will contribute to our understanding of severe respiratory infections in the elderly and will help to develop strategies to prevent or cure such severe infections.
